# Recent Advances in Assembly of Complex Plant Genomes

**DOI:** 10.1016/j.gpb.2023.04.004

**Published:** 2023-04-25

**Authors:** Weilong Kong, Yibin Wang, Shengcheng Zhang, Jiaxin Yu, Xingtan Zhang

**Affiliations:** Shenzhen Branch, Guangdong Laboratory for Lingnan Modern Agriculture, Genome Analysis Laboratory of the Ministry of Agriculture, Agricultural Genomics Institute at Shenzhen, Chinese Academy of Agricultural Sciences, Shenzhen 518120, China

**Keywords:** Complex plant genome, Assembly algorithm, Telomere-to-telomere genome, Haplotype-resolved assembly, Sequencing technology

## Abstract

Over the past 20 years, tremendous advances in sequencing technologies and computational algorithms have spurred plant genomic research into a thriving era with hundreds of genomes decoded already, ranging from those of nonvascular plants to those of flowering plants. However, **complex plant genome** assembly is still challenging and remains difficult to fully resolve with conventional sequencing and assembly methods due to high heterozygosity, highly repetitive sequences, or high ploidy characteristics of complex genomes. Herein, we summarize the challenges of and advances in complex plant genome assembly, including feasible experimental strategies, upgrades to **sequencing technology**, existing assembly methods, and different phasing algorithms. Moreover, we list actual cases of complex genome projects for readers to refer to and draw upon to solve future problems related to complex genomes. Finally, we expect that the accurate, gapless, telomere-to-telomere, and fully phased assembly of complex plant genomes could soon become routine.

## Introduction

High-quality genome assembly establishes a reference for exploring the evolutionary history and genetic mechanisms of complex traits and facilitates molecular breeding and genomics studies. Fast-growing sequencing technologies and algorithm innovations have promoted breakthroughs in both animal and plant genomes to date. However, assemblies of plant genomes are much more challenging than those of animal genomes, because most animal genomes are diploid and contain fewer repetitive sequences compared to plant genomes [1], [2]. In contrast, plant genomes span several orders of magnitude in size, vary in ploidy and heterozygosity levels, and contain a large number of different types of repeats (35%–90% of the genome) [3]. Since the first plant genome release for the model plant *Arabidopsis thaliana* in 2000 [4], more than 800 plant genomes have been published to date, including the genomes of eudicots, monocots, gymnosperms, ferns, lycophytes, bryophytes, charophytes, and chlorophytes [5]. However, most of these published plant genomes are simple genomes characterized by < 0.8% heterozygosity, and/or < 60% repetitive sequences, whereas chromosome-scale assemblies of plant genomes with highly repetitive sequences, high heterozygosity, or polyploid genomes are incredibly scarce.

Resolving highly repetitive sequences is meaningful for understanding genome evolution and for mining functional elements. For instance, many plants are dioecious with a newly evolved Y chromosome. The suppressed recombination region in the Y chromosome accumulates a large number of mobile elements, which could account for more than 90% of the examined region [6], [7], [8]. Although some sex determination factors have been identified in a few plant species, including papaya [9], poplar [10], and fig trees [11], the assembly of highly repetitive sequences poses notable challenges, hindering the discovery of sex determination mechanisms in a massive number of dioecious plants. In addition, nearly 80% of plants have undergone whole-genome duplication(s), and many of them still maintain polyploidy with a high level of heterozygosity among haplotypes [1]. Polyploidy is considered the main force of plant evolution [2], contributing to many well-known crops that humans rely on for survival, including wheat (*Triticum aestivum*), rape (*Brassica napus*), upland cotton (*Gossypium hirsutum*), peanut (*Arachis hypogaea*), strawberry (*Fragaria ananassa*), potato (*Solanum tuberosum*), banana (*Musa* spp.), and sugarcane (*Saccharum officinarum*).

Given the importance of these plant species, complex plant genome sequencing has been an emerging frontier in the genomics field. Recently, single-molecule sequencing (SMS) technologies and haplotype assembly algorithms have efficiently generated chromosome-scale and haplotype-phased complex genome assemblies for a few species, including potato [12], [13], [14], [15], [16], sugarcane [17], [18], and alfalfa [19]. Herein, we summarize the challenges of complex genome assembly, the advantages of SMS platforms, and newly developed assembly algorithms in complex plant genome assembly, aiming to provide a comprehensive reference facilitating future genome projects.

## Challenges of complex genome assembly

### High repetitive sequence content

Repetitive sequences that are similar or identical to sequences elsewhere in the genome represent an important and pervasive part of the dark matter of genomes [20], [21]. Many plant genomes are filled with repetitive sequences, including various satellites, rDNA, short interspersed nuclear elements, long interspersed nuclear elements, long terminal repeat (LTR) retrotransposons, and DNA transposons [22]. For instance, the total repetitive sequences account for ∼ 85% in maize genome [23] and in the wheat genome [24], and 74%–80% in the tea plant genome [25]. These different types of repetitive sequences contain anywhere from two copies to millions of copies, ranging from 1–2 bases (mono- and dinucleotide repeats) to millions of bases [20], [26]. These repeat-rich regions usually involve many important genetic functional regions, namely, telomeres, centromeres, multicopy genes, and non-recombining and highly heterochromatic chromosomes such as the Y and W sex chromosomes [11], [27]. Given that these regions play essential roles in the function and evolution of the genome [28], [29], [30], [31], the need to precisely assemble them has become a hurdle in complex genome studies.

**Figure 1:**
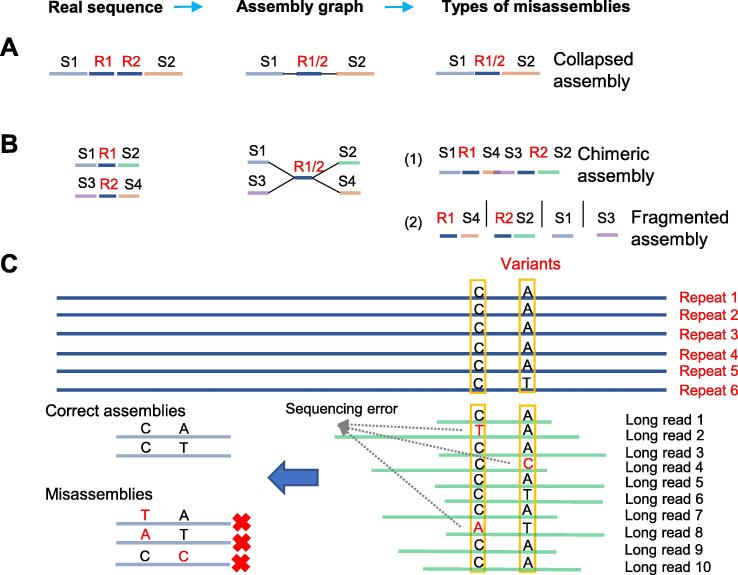
**The assembly of highly repetitive sequences** **A.** A collapsed assembly error example in tandem repeats. A tandem repeat containing two copies (R1 and R2) separates unique sequences S1 and S2. **B.** Chimeric or fragmented assembly errors in long segmental repeats among different chromosomal regions. S1, S2, S3, and S4 indicate unique sequences, and R1 and R2 represent two identical long segmental repeats. **C.** The impact of sequencing errors on the assembly of highly similar repeats.

However, repetitive sequences with hundreds or thousands of repeat units are widely distributed in genomes and cover an ultralong genomic region (such as nest LTRs, which can span 20–200 kb) that cannot be spanned by even long reads generated by SMS. In assemblies with short reads (35–800 bp) as input, nearly identical tandem repeats usually fold into fewer copies (*i.e.*, collapsed assembly, Figure 1A), making it difficult to determine the true number of copies. Similarly, unzipping two identical interspersed repeat units from the assembly graph can produce false joins with flanking unique sequences, leading to chimeric and fragmented contigs (Figure 1B). Due to improvements in the length of SMS reads, many repeat regions can now be well resolved, except for the extremely long and complex repeat regions. However, the high level of sequencing errors in SMS reads poses challenges to accurately distinguish minor variants among repetitive sequences from sequencing errors. On the one hand, low-frequency genetic variants with frequencies lower than the sequencing error rate may be mistaken for sequencing error, leading to the underestimation of low-frequency genetic variations. On the other hand, the sequencing error will be mistaken for the genetic variants, resulting in misassembly when sequencing errors are not corrected (Figure 1C).

### High heterozygosity

**Figure 2:**
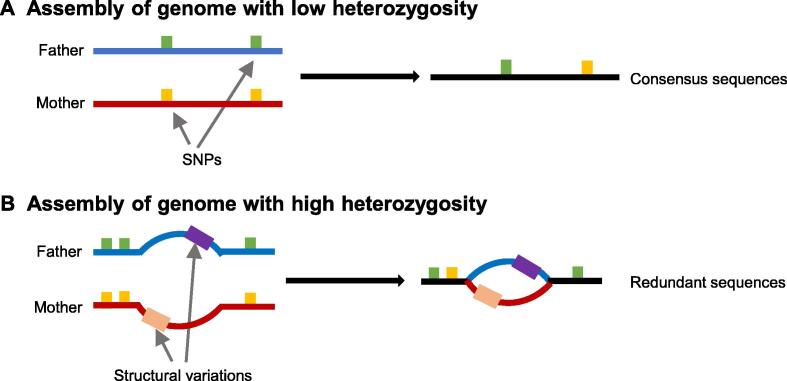
**Theimpact of high heterozygosityon genome assembly** **A.** Consensus sequence assembly of a low-heterozygosity genome. Small-scale variations (such as SNPs) in different haplotype sequences can be aligned during assembly and then assembled into consensus sequences. **B.** Bubble structures of highly heterozygous genomes. Large-scale structural variations from different haplotype sequences affect the sequence alignment to form ‘bubbles’ representing redundant allelic sequences and fail to form consensus sequences. SNP, single-nucleotide polymorphism.

In low-heterozygosity species, the variations between the two haplotypes are mainly small-scale. These small-scale variations enable accurate alignment during assembly, resulting in consensus sequences (Figure 2A). However, genomes with high heterozygosity contain many large-scale structural variations between the two haplotypes, leading to assembly ‘bubbles’ that represent redundant allelic sequences (Figure 2B). Many plants have high genome heterozygosity due to distant hybridization and self-incompatibility. Therefore, the assembly of these highly heterozygous genomes usually generates a larger size than the estimated size of the haploid genome.

### Polyploidy

Plant polyploidizations originate either from whole-genome duplication of a single species (autopolyploidy) or interspecific hybridization followed by chromosome doubling (allopolyploidy) [32], [33]. The assembly of allopolyploid genomes is less complex, and the first wave of polyploid genome assemblies has mainly involved allopolyploid crops, such as rape (*B. napus*), cotton (*G. hirsutum*), and peanut (*A*. *hypogaea*). It is relatively easy to distinguish subgenomes originating from different ancestral species because they have maintained a large proportion of genetic variations during their long evolutionary history. However, autopolyploid organisms consisting of more than two homologous sets of chromosomes pose significant challenges in genome assembly and haplotype phasing due to the high similarity between homologous chromosomes [34].

**Figure 3:**
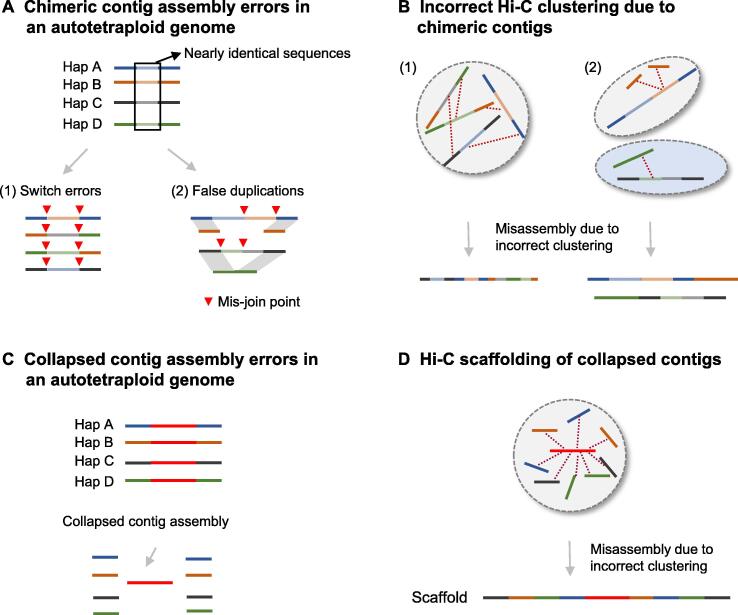
**Challenges of polyploid genome assembly** **A.** Illustration of chimeric contig assembly errors in an autotetraploid genome, including switch errors and false duplications. **B.** Incorrect Hi-C clustering of chimeric contigs leads to multiple misassemblies. **C.** Illustration of collapsed contig assembly errors in an autotetraploid genome. **D.** The collapsed contig generates Hi-C links with all contigs belonging to four haplotypes, resulting in a superlong and erroneous scaffold. Hap, haplotype; Hi-C, high-throughput/resolution chromosome conformation capture.

For example, the autotetraploid genome has four similar haplotypes that contain a large proportion of nearly identical sequences. Linking these identical sequences has a tendency to generate chimeric contigs with switch errors or false duplications (Figure 3A). These chimeric contigs confuse high-throughput/resolution chromosome conformation capture (Hi-C) signals, resulting in erroneous scaffolds that mess up sequences from different haplotypes (Figure 3B). In addition, nearly identical homologous sequences between different haplotypes cannot be accurately distinguished, leaving many collapsed contigs (Figure 3C). Furthermore, these collapsed contigs generate Hi-C links with phased contigs belonging to different haplotypes, resulting in superlong and erroneous scaffolds (Figure 3D).

## Technical innovations in complex genome assembly

### Evolution of sequencing platforms

Earlier plant genome assemblies were generated using Sanger sequencing and next-generation sequencing (NGS) technologies combined with the minimum tiling path, the overlap layout consensus, or *de Bruijn* graph approaches [35], [36], [37], [38] for species such as *A*. *thaliana* [4], *Oryza sativa* [39], [40], *Carica papaya* [41], *G. max* [42], and *Populus trichocarpa* [43]. Although widely used in many genome projects, these sequencing technologies have limited power to overcome assembly challenges in complex genomes due to the limited length of short reads (< 1000 bp) and inevitable GC bias. For instance, sequencing a 2.3-Gb maize genome relied on the construction of 16,848 bacterial artificial chromosome (BAC) libraries. This process was highly labor intensive and generated a fragmented assembly with more than 10% of genomic sequences missing [23].

The subsequent SMS technologies advanced by Pacific Biosciences (*i.e.*, PacBio) and Oxford Nanopore Technology (ONT) companies were able to generate long reads that could span the kilobase- or even megabase-level repetitive regions along chromosomes. The first plant genome (*Oropetium thomaeum*) assembled based on only PacBio long reads demonstrated the ability of genome assembly in terms of contiguity and completeness [37], [44]. In addition, the maize B73 genome assembled by PacBio data had a 52-fold increase in contig continuity with reduced assembly errors in the centromeric region compared with the previous version [23] and greatly facilitated the annotation of functional genes and the evolutionary analysis of transposons [45]. Although SMS technologies have made revolutionary advancements in the assembly of complex plant genomes, they suffer from a higher sequencing error rate, ranging from 5% to 20% [46]. To address this issue, PacBio adopts the circular consensus sequencing model to generate long high-fidelity (HiFi) reads by reading multiple passes of a single template molecule [47]. This strategy achieves a read accuracy of more than 99.8% but at the cost of read length.

### Experimental approaches for genome scaffolding

The reconstruction of chromosomes is an ultimate goal in genome assembly, aiming to determine the orientation and orders of contigs globally. This step, called scaffolding, is vital for many downstream analyses and applied tasks, including the identification of genome-wide genotype–phenotype associations, marker-assisted breeding, and chromosome evolution analysis. Genetic maps were widely applied to early genome projects for genome scaffolding, such as *Arabidopsis* [4] and rice genomes [39], [40]. It successfully solved some complex genome assemblies, including that of the hexaploid bread wheat genome [48].

During the past decade, fruitful achievements have been made in experimental approaches for genome scaffolding, including BioNano optical maps using a light microscope-based technique to capture the physical locations of selected enzymes and a chromatin conformation capture technique (Hi-C) based on proximity ligation of chromatin. These two novel scaffolding approaches can quickly and accurately reconstruct the chromosomes for some complex plant genomes. Despite the limitation of sparse enzyme sites and the requirement of extraction of long DNA molecules [49], BioNano technology has shown its power in chromosomal-scale genome assembly in some plant genome projects, such as that of sorghum [50]. Hi-C technology can construct linkage information between contigs by detecting long-distance DNA interactions and has become routine for most genome projects. Applying Hi-C has resulted in the successful assembly of dozens or even hundreds of genomes, especially some complex polyploid genomes, such as those of sugarcane [17] and alfalfa [51].

## Strategies for monoploid assembly in diploid genomes

Most diploid genome projects aim to generate ‘consensus’ sequences (*i.e.*, monoploid assembly) to represent a reference genome for a given species (Figure 2A). This goal can be easily achieved for some plant genomes with extremely low heterozygosity, such as those of *Arabidopsis* and rice. However, the assembly of heterozygous genomes requires additional processes to solve ‘bubbles’ representing redundant sequences in initial contigs, which contain a large proportion of allelic contigs that originate from homologous chromosomes. Thus, three main strategies have been designed to classify these redundant contigs: read depth (RD)-, whole genome alignment comparison (WGAC)-, and *K*-mer-based strategies.

**Figure 4:**
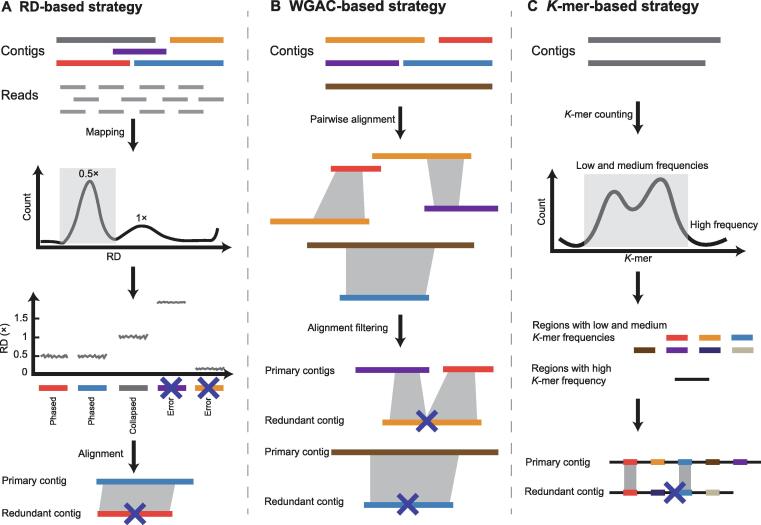
**Three strategies for identifying redundant contigs** **A.** With the RD-based strategy, redundant or phased contigs are approximately one-half of the mapped RD of collapsed or haplotype-fused contigs due to the bisected RD and the extreme similarity between redundant contigs. Based on the RD of contigs, phased contigs and collapsed contigs can be accurately identified, and the redundant phased contigs will be filtered. **B.** With WGAC-based strategy, contigs with long-scale alignment are identified as redundant contigs, and only the longer one is selected to leave in the monoploid genome. **C.** In the *K*-mer-based strategy, more than 40× Illumina or BGI short reads are first used to build the *K*-mer data pool. Then, low- and medium-frequency *K*-mers are mapped to assembled contigs. Redundant contigs share a high proportion of low- and medium-frequency *K*-mers, and relatively long contigs are finally selected to leave in the monoploid genome. RD, read depth; WGAC, whole genome alignment comparison.

The RD-based strategy identifies collapsed and redundant sequences by investigating the sequencing depth of mapped reads. The RDplot of the initial contigs in a highly heterozygous genome usually shows a bimodal distribution. Suppose collapsed or haplotype-fused contigs have a 1× RD. In that case, redundant contigs will only have approximately 0.5× RD, because redundant sequences will evenly distribute total reads due to the sequence similarity of two redundant contigs (Figure 4A). As a typical example, purge_haplotigs software [52] utilizes the RD-based strategy for identifying and removing these redundant sequences from a heterozygous assembly and eventually retains primary contigs to construct the monoploid genome (Figure 4A). The RD-based strategy has been successfully applied to several highly heterozygous genomes, namely, golden buckwheat (*Fagopyrum dibotrys*) [53], red clover (*Trifolium pratense* L.) [54], lilacs (*Syringa oblata* L.) [55], and carnation (*Dianthus caryophyllus*) [56]. However, the RD-based strategy consumes time and money due to the need for large-scale global alignment of genome sequences.

In contrast, several software programs, such as Pseudohaploid [19], purge_dups [57], and Redundans [58], implement the WGAC-based strategy to identify allelic contigs that have a high level of similarity and overlapping sequences. The long alignment chains detected by pairwise comparison between assembled contigs are considered redundant homologous regions. Only one copy of these homologous regions with a longer size was eventually retained as a representative haplotype (Figure 4B). However, the WGAC-based strategy is also time-consuming due to the global contig pairwise comparison.

To efficiently detect redundant contigs in complex genomes, a *K*-mer-based strategy named Khaper was proposed [46]. The basic concept of Khaper is to search for common low- and medium-frequency *K*-mers via pairwise comparison between contigs and to identify potential allelic contigs if they share a high proportion of low- and medium-frequency *K*-mers (Figure 4C). Because it does not rely on genome-wide sequence alignment, Khaper significantly saves central processing unit (CPU) time and solves the problems of time consumption and overutilization of computational resources in removing the redundancy process of large genomes with high heterozygosity [46].

## Toward haplotype-resolved assembly

Most reference genomes for diploid and polyploid organisms stay at the ‘monoploid’ level, which represents ‘mosaic’ sequences that mix two or more homologous chromosomes. Unzipping accurate haplotypes in a polyploid genome is beset with difficulties. In the case of an *n*-ploid organism, *n* − 1 haplotypes must be computed before the haplotype of interest can be inferred. For a pair of single-nucleotide polymorphisms (SNPs) in a polyploid, there are theoretically *n*! connection possibilities. Recently, upgraded sequencing technologies and innovations in algorithms and strategies have provided the basis for assembling highly heterozygous diploid and polyploid genomes at the haplotype level rather than at the monoploid level.

To date, the built-in heuristic algorithms of multiple reference-based or *de novo* phasing tools have been systematically summarized [34], [59], [60]. However, many have been developed for the human genome and have not been applied well to plant genome assembly. Here, to explore effectual phased tools and strategies for assembling complex plant genomes, we have summarized the recently published haplotype-resolved assemblies in plant genomes. We have further divided these assembly approaches into two strategies: reference-based variant phasing and *de novo* assembly-based haplotype phasing (Table 1) [12], [51], [14], [15], [16], [17], [18], [61], [62], [63], [64], [65], [66], [67], [68], [69], [70], [71], [72], [73], [74], [75], [76], [77], [78], [79], [80], [81], [82], [83], [84], [85], [86], [87], [88], [89], [90].

**Table 1:** **Summary of available haplotype-resolved plant genome assemblies**

**Phasing strategy**	**Species**	**Karyotype**	**Sequencing platform**	**Toolor strategy**	**Ref.**
**Reference-based variant phasing**					
	*Litchi chinensis*	2*n* = 2*x* = 30	Illumina + PacBio + 10X Genomics	HapCUT2	[61]
	*Ipomoea batatas*	2*n* = 6*x* = 90	Illumina	Ranbow	[62]
***De novo*assembly-based haplotype phasing**					
*De novo* phased contig tools + Hi-C scaffolding	*Bletilla striata*	2*n* = 2*x* = 32	HiFi + Hi-C	HiFiasm + LACHESIS	[63]
	*Bupleurum chinense*	2*n* = 2*x* = 12	Illumina + HiFi + Hi-C	HiFiasm + 3D-DNA	[64]
	*Suaeda glauca*	2*n* = 2*x* = 18	HiFi + Hi-C	HiFiasm + 3D-DNA	[65]
	*Cynodon dactylon*	2*n* = 4*x* = 36	Illumina + PacBio + Bionano + Hi-C	HiFiasm + 3D-DNA	[66]
	*Populus tomentosa*	2*n* = 3*x* = 57	Illumina + HiFi + Hi-C	HiFiasm + 3D-DNA	[67]
	*Malus domestica*	2*n* = 2*x* = 34	Illumina + 10X Genomics + HiFi	HiFiasm + DeNovoMAGIC	[68]
	*Manihot esculenta*	2*n* = 2*x* = 36	Illumina + HiFi + Hi-C	HiFiasm + ALLHiC	[69]
	*Pogostemon cablin*	2*n* = 4*x* = 64	Illumina + PacBio + Hi-C	Canu + 3D-DNA	[70]
	*Manihot esculenta*	2*n* = 2*x* = 36	Illumina + PacBio + Hi-C	FALCON + FALCON_unzip + FALCON-Phase	[71]
	*Humulus lupulus*	2*n* = 2*x* = 20	Illumina + PacBio + Hi-C	FALCON + FALCON-unzip	[72]
	*Vanilla planifolia*	2*n* = 2*x* = 28	Illumina + ONT	Miniasm + FALCON-Phase + LACHESIS	[73]
	*Hydrangea macrophylla*	2*n* = 2*x* = 36	Illumina + PacBio + Hi-C	FALCON + FALCON_unzip + FALCON-Phase	[74]
	*Zingiber officinale*	2*n* = 2*x* = 22	Illumina + PacBio + Hi-C	FALCON-Phase + 3D-DNA	[75]
	*Saccharum spontaneum*	1*n* = 4*x* = 32	Illumina + BACs + PacBio + Hi-C	Canu + ALLHiC	[17]
	*Saccharum spontaneum*	2*n* = 4*x* = 40	HiFi + Hi-C	Canu + ALLHiC + 3D-DNA	[18]
	*Camellia sinensis*	2*n* = 2*x* = 30	HiFi + Hi-C	HiFiasm + ALLHiC	[76]
	*Solanum tuberosum*	2*n* = 4*x* = 48	HiFi + Hi-C	HiFiasm + ALLHiC	[15]
	*Camellia sinensis*	2*n* = 2*x* = 30	Illumina + PacBio + Hi-C	Canu + ALLHiC	[77]
	*Manihot esculenta*	2*n* = 2*x* = 36	PacBio + Hi-C	Canu + Wtdbg + ALLHiC	[78]
	*Dendrocalamus latiflorus*	2*n* = 6*x* = 70	Illumina + PacBio + Hi-C	Falcon + ALLHiC	[79]
	*Medicago sativa*	2*n* = 4*x* = 32	Illumina + HiFi + Hi-C	Canu + ALLHiC	[51]
	*Medicago sativa*	2*n* = 4*x* = 32	PacBio + Bionano + Hi-C	Canu + MECAT + ALLHiC	[80]
	*Medicago sativa*	2*n* = 4*x* = 32	PacBio + Hi-C	Canu + ALLHiC	[81]
	*Artemisia annua*	2*n* = 2*x* = 18	Illumina + PacBio + Bionano + Hi-C	Canu + HiFiasm + FALCON + LACHESIS	[82]
Trio-binning-based *de novo* phasing	*Ananas comosus*	2*n* = 2*x* = 50	PacBio + ONT + Hi-C + Illumina	Trio-binning	[83]
	*Cerasus* × *kanzakura*	2*n* = 2*x* = 16	Illumina + PacBio	Trio-binning	[84]
	*Cerasus* × *yedoensis*	2*n* = 2*x* = 16	Illumina + PacBio	Trio-binning	[85]
Genetic map-based *de novo* phasing	*Pyrus bretschneideri*	2*n* = 2*x* = 34	BACs + SCS	Gamete binning	[86]
	*Solanum tuberosum*	2*n* = 4*x* = 48	HiFi + SCS	Gamete binning	[16]
	*Camellia sinensis*	2*n* = 2*x* = 30	SCS	Gamete binning	[87]
	*Prunus armeniaca*	2*n* = 2*x* = 16	PacBio + SCS	Gamete binning	[88]
	*Solanum tuberosum*	2*n* = 2*x* = 24	ONT + 10X Genomics + HiFi + Hi-C + Illumina for self-population	Population resequencing	[12]
	*Solanum tuberosum*	2*n* = 4*x* = 48	HiFi + Hi-C + Illumina for self-population	Population resequencing	[14]
	*Vitis riparia*	2*n* = 2*x* = 38	Illumina + PacBio + 10X Genomics + GBS data	Population resequencing	[89]
	*Zoysia japonica*	2*n* = 4*x* = 20	PacBio + GBS data	Population resequencing (PolyGembler)	[90]

*Note*: Hi-C, high-throughput/resolution chromosome conformation capture; PacBio, Pacific Biosciences; ONT, Oxford Nanopore Technologies; GBS, genotyping-by-sequencing; BAC, bacterial artificial chromosome; SCS, single-cell sequencing; HiFi, high fidelity.

### Reference-based variant phasing

In reference-based variant phasing, a high-quality genome is required as the reference to distinguish different haplotypes based on long-range linked allelic variants through alignments of sequencing reads against the reference genome using different phasing algorithms, including minimum error correction [91], weighted minimum letter flip [59], maximum fragment cut [92], and polyploid balanced optimal partition [93] as different frameworks [34], [59], [60]. More than 20 reference-based variant phasing tools have been developed (Table S1). However, only HapCUT2 [94] and Ranbow [95] were effectively used in haplotype-resolved genome assemblies of *Litchi chinensis* (diploid) [61] and *Ipomoea batatas* (hexaploid) [62] (Table 1). HapCUT2 utilizes advanced hybrid phasing programs and can handle chromosome-scale phasing using multiple types of sequencing data, including HiFi, 10X Genomics linked reads, and Hi-C reads [94]. In contrast, Ranbow is designed for haplotype reconstruction of the polyploid genome using a graph-based algorithm and can integrate all types of small variants in bi- and multiallelic sites to reconstruct haplotypes [95]. Although the reference-based variant phasing strategy shows its ability with less computational consumption, it also has drawbacks that limit its application to a wide range of complex genomes. The accuracy of this strategy is affected by a series of factors, including the quality of the reference genome, read length, sequencing depth, sequencing errors, and repeats. For instance, most plant genomes contain a large proportion of repetitive sequences, leading to ambiguous read mapping and inaccurate identification of variants. In addition, reference-based variant phasing tools mostly ignore large-scale allelic variants due to the inefficacy of identifying structural variations based on read mapping.

### *De novo* assembly-based haplotype phasing

In contrast to reference-based variant phasing, which mainly retains single-nucleotide allelic variants, *de novo* assembly-based haplotype phasing tends to be more comprehensive. It can produce a noncollapsed haplotype-phased assembly, covering large types of allelic variants, such as indels and structural variants [34].

Most of the phased plant genomes published to date were completed by relying on *de novo* phased contigs followed by Hi-C scaffolding (*i.e.*, *de novo* phased contig tools + Hi-C scaffolding). Briefly, allelic contigs are initially assembled and phased by allele-aware algorithms implemented in PacBio assemblers (*e.g.*, Hifiasm [96], [97], Canu [98], and FALCON-Unzip [99]). The phased contigs are subsequently subjected to Hi-C scaffolding tools (such as LACHESIS [100], 3D-DNA [101], FALCON-Phase [102], and ALLHiC [103]), achieving haplotype construction at the chromosome level (Table 1). Hifiasm and Canu use haplotype-aware graphs with reads as nodes and read overlaps as edges to assemble all contigs from different haplotypes [97], [98]. In heterozygous diploid genomes, Hifiasm can solve haplotype-aware graphs based on Hi-C reads that provide long-range links between contigs, in which step allelic contigs are fully separated into two haplotypes [96], [97]. In contrast, Canu requires postprocessing to assign contigs to haplotypes with tools such as Purge_dups [57], FALCON-Phase [102], and ALLHiC [103] to split contigs into different haplotypes [98]. The widely used Hi-C scaffolding programs in haplotype-resolved genome assembly include 3D-DNA and ALLHiC. Benefiting from the fully separated allelic contigs in Hifiasm, 3D-DNA takes contigs from each haplotype as inputs and implements scaffolding algorithms in highly homozygous diploid genomes. However, it has limited power to work with the assembled allelic contigs that are not separated into haplotypes in the polyploid genomes. ALLHiC uses a novel pruning step to remove Hi-C links between phased contigs and collapsed regions as well as allelic Hi-C signals based on a customized allelic contig table [103]. Removing the interference of Hi-C links allows the phased contigs to be accurately partitioned according to the strength of the Hi-C links. However, ALLHiC depends heavily on the initial assembly quality, a phenomenon that is known as “garbage in, garbage out” [103].

Recently, trio-binning-based diploid phasing algorithms for trio sequencing data have been developed, including TrioCanu [104], Hifiasm + trio [97], and WHdenovo [105]. The long sequencing reads of an F_1_ hybrid with a high level of heterozygosity are first partitioned into paternal and maternal read sets based on the unique parental Kmers [104]. The two read sets are assembled separately into two haploid genomes, with each representing a parental genome. Although trio-binning-based algorithms perform exceptionally well in terms of continuity and accuracy of phased contigs, they have limited application to complex plant genomes due to a lack of parental data. In addition, genomic regions that are heterozygous in both parents cannot be phased [34].

Genetic maps have been widely used in early genome projects for chromosome construction. Additionally, they demonstrate an ability to carry out haplotype phasing by resequencing hundreds of individuals in a derived population (*e.g.*, a selfing population). In a heterozygous diploid potato (RH), all contigs were assembled from high-quality long reads and 10X Genomics linked reads. Then, each contig was regarded as a molecular marker, and the copy number (0, 1, 2) of the contig in each progeny was inferred based on the distribution of each individual read number, corresponding to the genotype (aa, Aa, AA). The genotype matrix of all contigs in the selfing population allowed the contigs to be divided into 24 linkage groups corresponding to 12 chromosome pairs using traditional genetic mapping strategies. Finally, the long reads and 10X Genomics linked reads for each linkage group were retrieved and reassembled to generate an improved scaffold assembly [12]. Bao et al. recently introduced this approach into the tetraploid potato genome and assembled the haplotype-resolved genome of a tetraploid cultivated potato [14]. In addition, gamete binning, a method based on single-cell sequencing of hundreds of haploid gamete genomes, enables the separation of long sequencing reads (such as HiFi reads) into two haploid-specific read sets. After the independent assembly of reads for each haplotype, contigs were scaffolded to the chromosomal level using a genetic map derived from recombination patterns within the same gamete genomes [88]. Gamete binning has been efficiently used to infer genome-wide haplotypes in diploid pear, apricot tree, tea plant, and tetraploid potato [106], [86], [87], [88]. However, the construction of the genetic map relies on extensive meiotic recombination, which often means genotyping hundreds of recombined genomes, leading to doubling of sequencing costs relative to other strategies. Moreover, the separation of gametes is severely limited by the level of the experimental technique and by specific seasons. In addition, developing derived populations is time-consuming and costly and may pose significant challenges if the individuals show long juvenility or sterility [17].

## Implications of haplotype-resolved genome assembly

In the era of NGS, the compromise method is to use sequence-derived haploid materials or to tolerate chimeric heterozygous regions to construct a reference genome. Therefore, most reference genomes for diploid and polyploid organisms stay at the ‘monoploid’ level, which represents ‘mosaic’ sequences from more than two homologous chromosomes yet fails to capture allelic variants that are diploid or polyploid in nature and that may be associated with compound heterozygotes, dosage effects, homeolog silencing, heterosis, population genetics, and species evolution [34]. The functional and evolutionary study of polyploids would require a full dissection of the different allele sequences. Accurate haplotype-resolved genome assembly is essential for analyzing haplotypic structural variants and allele-specific expression for complex traits, such as heterosis and genomic imprinting. Additionally, a better understanding of haplotypic variations is key to designing advanced breeding strategies, especially for overcoming severe inbreeding depression or for improving crop yield. Recently, several research groups have generated haplotype-resolved genome assemblies for several important plant species, including sugarcane [17], banyan tree [11], tea plant [77], and potato [12], [13], [14], [15], [16]. These studies not only established references for the assembly of complex genomes but also provided new insights into genome evolution and biological questions concerning these horticulture or crop plants.

The application of the most advanced SMS sequencing and Hi-C technologies has successfully anchored an autotetraploid sugarcane genome onto 32 chromosomes [17]. Based on a high-quality phased genome, a syntenic analysis confirmed two rounds of whole-genome duplication events in the sugarcane species. The reduction in chromosome bases from 10 to 8 in *Saccharum spontaneum* compared with sorghum has resulted from two chromosome fissions and two fusions.

The Tieguanyin tea cultivar has been cultivated for approximately 300 years, and its genome accumulates a large number of somatic mutations, including deleterious mutations, during the long-term asexual reproduction process. This process increases the genetic load and reduces adaptability. However, our knowledge of dealing with genetic load in the context of vegetatively propagated crops is limited. A fully phased assembly genome provides two sets of alleles that allow the precise study of the allele-specific expression pattern in multiple tissues. The authors found that asexually propagated individuals prefer ancestral or beneficial alleles rather than deleterious mutations to maintain plant growth and development as well as adaptability to the environment [77].

Potato (*S*. *tuberosum* L.) is one of the most important tuber crops, but its genetic improvement is slow due to tetrasomic inheritance and clonal propagation [16]. Asexual propagation through tubers is prone to the accumulation of deleterious mutations and has a higher cost than seed propagation [107]. However, the decline in selfing caused by deleterious mutations is an obstacle in potato seed propagation. To address this problem, Zhou et al. identified dispersed deleterious mutations and differentially expressed alleles based on a phase diploid genome, which provides operational targets for eradicating harmful alleles or for the accumulation of beneficial alleles through recombination [12]. This study has subsequently resulted in a breakthrough in potato breeding, leading to vigorous inbred-line-based F_1_ hybrids with strong heterosis [107]. Based on the haplotype-resolved genome of a tetraploid potato cultivar (Otava), Sun et al. found that only 53.6% of the genes have all four haplotypes, and some of the four haplotypes for one gene are identical. Thus, there are only 3.2 haplotypes and 1.9 distinct alleles per gene, suggesting that potato yield and resistance can still be further improved by increasing the allelic diversity of the tetraploid genome because heterosis itself is based on nonadditive interactions of different alleles [16]. In addition, there were benefits from the phased assembly of tetraploid potato; deleterious mutations between homologous chromosomes were systematically identified; and the mutual shielding of deleterious mutations and functional gene complementation between parents were further reported [14]. In another phased assembly report on tetraploid potato, researchers analyzed the number and roles of deleterious and dysfunctional genes in the four haplotypes across six tetraploid cultivated potatoes. The autotetraploid potato is a clonally propagated species that undergoes limited meiosis. Its dysfunctional and harmful alleles are not eliminated, which significantly increases the difficulty of breeding. Using phased deleterious and dysfunctional gene information will help breeders create the best allele combination in the F_1_ potato generation, thereby improving potato yield and quality [13].

*Ficus hispida* is a dioecious plant species, but its sex determination mechanism is a mystery owing to the lack of assembly of sex chromosomes, making it impossible to directly compare the sequence differences between the X and Y chromosomes. To investigate sex determination, our previous study on *Ficus* genomes proposed a novel pipeline, sex phase, to separate X and Y chromosomes by utilizing resequencing reads from individuals of known sex [11]. The comparative analysis of the phased sex chromosomes highlighted important structural variations between the X and Y chromosomes, including chromosome size (22.6 Mb in Y *vs.* 21.9 Mb in X) and a genomic inversion between 0.61 Mb and 1.57 Mb on the Y chromosome. Importantly, one protein-coding gene, *AGAMOUS 2* (*AG2*), in the sex-determining region, whose ortholog is essential for the development of the stamen and the carpel in *Arabidopsis* [108]*,* is present in male individuals but absent in females. Furthermore, PCR amplification of three representative species of the dioecious subgenera confirmed that the *AG2* gene is likely a dominant sex-determining factor across the *Ficus* genus.

## The era of telomere-to-telomere assembly

A more challenging task is to generate gapless or telomere-to-telomere (T2T) assemblies of complex plant genomes. Recently, the goal of near-T2T assemblies has been achieved in rice [27], [109], [110], *Arabidopsis* [111], [112], [113], banana [114], and watermelon [115]. These near-T2T assemblies not only provide the opportunity to update the knowledge of megabase-scale tandemly repeated satellite arrays and epigenetic organization in centromeres, but also indicate that the plant genome has entered the T2T era [27], [111]. In sequencing techniques and assembly strategies, these near-T2T genomes all used recent assembly algorithms such as Hicanu and Hifiasm to complete primary contig assembly and fill gaps with high-precision HiFi reads. The remaining gaps were then filled twice with the superlong ONT reads or BAC reads, and the gap sequences were finally corrected and polished with HiFi reads. Alternatively, Rautiainen et al. developed a hybrid genome assembly pipeline (Verkko) for T2T assembly by integrating HiFi and ultralong ONT reads, showing its power to generate the T2T assembly for human genomes [116]. However, the accumulated experience in these model-like plants to complete the T2T assemblies of the complex plant genomes remains a long way to go before completely solving the high heterozygosity, high repeat sequence content, and high ploidy problems of complex plant genomes.

## Discussion and future prospects

Deciphering complex plant genomes is of significance in understanding the basic biological mechanisms, including the discovery of key genes or structural variants related to resistance [117], [118] and sex determination [11]. Additionally, benefitting from the development of phasing algorithms, comparative analysis between/among haplotypes in highly heterozygous or polyploid genomes identifies abundant allelic variations, which provide a genetic basis for studying fundamental biological questions about heterosis and allelic imbalance.

In this review, we describe some examples of complex plant genome projects and many tools being used to address the complexity of genome assembly. We recommend that ∼ 30× HiFi and ∼ 100× Hi-C sequencing are necessary for high-quality assemblies of complex genomes. Contigs with high continuity and accuracy can be assembled and phased by a widely used HiFi assembler, Hifiasm [96], [97]. Chromosomal-scale assembly can be achieved using a series of Hi-C scaffolders, including 3D-DNA for diploid genomes and ALLHiC for polyploid genomes. In addition, a significantly improved gapless or T2T assembly requires additional ultralong ONT reads based on several successful cases [27], [109], [110], [111], [112], [113]. If parents or derived population materials exist, resequencing of these materials can obviously improve haplotype results. However, there is no all-powerful method applicable to all genomes and the optimal genome assembly sometimes needs testing of different pipelines.

Indeed, dozens of plant species that are economically important have ultracomplex genomes, leaving their genomic sequences under ongoing development. A typical example is modern hybrid sugarcane (*Saccharum* spp. hybrids), a widely cultivated crop of sugar and bioenergy production [119]. The nuclear genome of modern hybrid sugarcane is composed of subgenomes originally from *S. officinarum* (an octoploid species, with a basic chromosome number of 10, 2*n* = 80) and *S. spontaneum* (varied ploidy levels between 5× and 16×, a basic chromosome number of 8, 2*n* = 40–128). The hybrid sugarcane genome is much more complicated due to the uneven inheritance of genetic materials from its progenitors through interspecific crosses and one or more subsequent backcrosses [120], [121]. Modern hybrid sugarcane has a basic chromosome number of 10. However, its complexity resides in the mixture of aneuploid and homo(eo)logous chromosomes, which results in 10 uneven homo(eo)logous chromosome groups of the modern hybrid sugarcane genome carrying a total number of chromosomes ranging from 100 to 130 [122], [123]. It means that there are 8–14 homo(eo)logous copies for most genes in the hybrid sugarcane genome [123], [124]. The state-of-the-art Hi-C scaffolding technology loses its power on ultracomplex genomes mostly owing to an extremely low level of uniquely mapped short reads. The recently proposed Pore-C method, which integrates single-molecule long-read sequencing and three-dimensional chromatin conformation capture technology, is able to detect multiway interactions among different genomic regions and distinguish highly similar genomic sequences [125]. The experimental innovation promises an effective approach to avoid multiple alignment in polyploid genomes, likely solving the ultracomplex sugarcane genome assembly.

## Competing interests

The authors have declared no competing interests.

## CRediT authorship contribution statement

**Weilong Kong:** Investigation, Methodology, Formal analysis, Visualization, Writing – original draft, Writing – review & editing. **Yibin Wang:** Formal analysis, Visualization. **Shengcheng Zhang:** Formal analysis, Visualization. **Jiaxin Yu:** Formal analysis. **Xingtan Zhang:** Conceptualization, Methodology, Resources, Writing – original draft, Writing – review & editing, Supervision. All authors have read and approved the final manuscript.

## Supplementary material

Supplementary Table S1 **Programs and approaches for haplotype-resolved assembly**
