# Supplementary material for: Recent Advances in Assembly of Complex Plant Genomes
Source: Genomics Proteomics Bioinformatics. 2023 Apr 25;21(3):427–39. doi: 10.1016/j.gpb.2023.04.004 (PMC10787022; doi:10.1016/j.gpb.2023.04.004)
Supplement: Supplementary Table S1 — Programs and approaches for haplotype-resolved assembly [file mmc1.docx]

**Table S1 Programs and approaches for haplotype-resolved assembly**

| **Program / approach** | **Data** | **Advantages** | **Limitations** |
| --- | --- | --- | --- |
| **Reference-based variants phasing** | | | |
| WhatsHap, HapCUT2, ProbHap, SHAPEIT3, SHAPEIT4 | Long reads, such as PacBio, Nanopore and / or Hi-C reads | Efficient to resolve the phase between nearby variants | Designed for diploidy; do not allow full haplotype resolution across entire chromosome. |
| HapCompass, HANDS, SDhaP, HapTree, H-Pop, PolyHarsh, TriPoly, SCGD-hap, AltHap, HaplotypeAssembler, WhatsHap-polyphase, Hap++, Hap10, nPhase | Long reads and / or short reads | Excellent performance for phasing in small variants | Affected by reference genome quality and sequencing quality; the inability to deal with large-scale mutations limits application on complex polyploids. |
| ***De novo* phased assembly** | | | |
| DipAsm, SDA, Falcon Unzip | Long reads and / or linked reads, Hi-C reads | Work well for genomes with low heterozygosity | Rely on consensus sequences; fail in regions or genomes with high repeat and heterozygosity rates |
| HiCanu, Hifiasm, SDip | Hifi reads, ultra-long reads, or Hi-C reads | High consensus accuracy and continuity | No chromosome-scale assembly |
| TrioCanu, hifiasm+trio, Whdenovo | Short reads (parents) and long reads (offspring) | Perform extremely well in continuity and accuracy | Require family sequencing |
